# 2-(4-Methyl­pyridin-2-yl)-4′,4′,6′,6′-tetra­kis­(pyrrolidin-1-yl)-1*H*,2*H*-spiro­[naphtho­[1,2-*e*][1,3,2]oxaza­phosphinine-3,2′-[1,3,5,2,4,6]tri­aza­triphosphinine]

**DOI:** 10.1107/S1600536813014220

**Published:** 2013-05-31

**Authors:** Muhammet Işıklan, Ömer Sonkaya, Tuncer Hökelek

**Affiliations:** a Department of Chemistry, Kırıkkale University, 71450 Kırıkkale, Turkey; b Department of Physics, Hacettepe University, 06800 Beytepe, Ankara, Turkey

## Abstract

In the title spiro-phosphazene derivative, C_33_H_46_N_9_OP_3_, the phosphazene and six-membered N/O rings are in flattened chair and twisted-boat conformations, respectively. The naphthalene ring system and the pyridine ring are oriented at a dihedral angle of 41.82 (4)°. In the crystal, weak C—H⋯O hydrogen bonds link the mol­ecules related by translation along the *a* axis into chains. C—H⋯π inter­actions aggregate these chains into layers parallel to the *ab* plane.

## Related literature

For *N*/*O*-donor-type bifunctional reagents used for the reaction of hexa­chloro­cyclo­triphosphazene giving *spiro* derivatives, see: Beşli *et al.* (2007 ▶); Işıklan *et al.* (2010 ▶, 2013 ▶). For bond-length data, see: Allen *et al.* (1987 ▶). For the standard compound, N_3_P_3_Cl_6_, see: Bullen (1971 ▶). For ring puckering parameters, see: Cremer & Pople (1975 ▶).
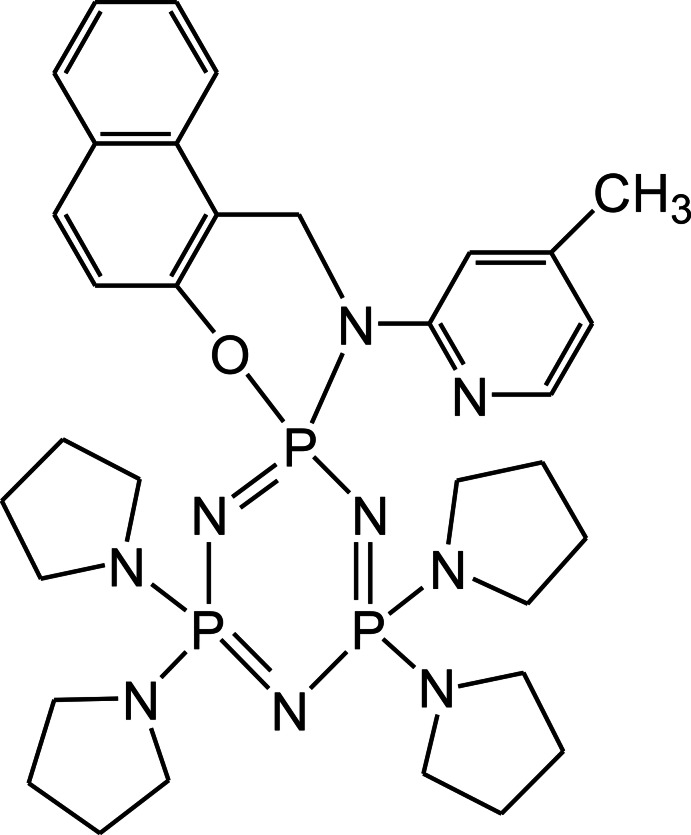


## Experimental

### 

#### Crystal data

C_33_H_46_N_9_OP_3_
*M* *_r_* = 677.70
Triclinic, 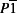

*a* = 9.5830 (2) Å
*b* = 10.9142 (2) Å
*c* = 16.8172 (3) Å
α = 79.210 (2)°
β = 84.542 (3)°
γ = 74.193 (2)°
*V* = 1660.68 (6) Å^3^
*Z* = 2
Mo *K*α radiation
μ = 0.22 mm^−1^
*T* = 100 K
0.31 × 0.26 × 0.25 mm

#### Data collection

Bruker Kappa APEXII CCD area-detector diffractometer
Absorption correction: multi-scan (*SADABS*; Bruker, 2005 ▶) *T*_min_ = 0.934, *T*_max_ = 0.947
30003 measured reflections
8270 independent reflections
6876 reflections with *I* > 2σ(*I*)
*R*_int_ = 0.030

#### Refinement

*R*[*F* ^2^ > 2σ(*F* ^2^)] = 0.040
*wR*(*F* ^2^) = 0.109
*S* = 1.04
8270 reflections
416 parameters
H-atom parameters constrained
Δρ_max_ = 0.89 e Å^−3^
Δρ_min_ = −0.37 e Å^−3^

### 

Data collection: *APEX2* (Bruker, 2007 ▶); cell refinement: *SAINT* (Bruker, 2007 ▶); data reduction: *SAINT*; program(s) used to solve structure: *SHELXS97* (Sheldrick, 2008 ▶); program(s) used to refine structure: *SHELXL97* (Sheldrick, 2008 ▶); molecular graphics: *ORTEP-3 for Windows* (Farrugia, 2012 ▶); software used to prepare material for publication: *WinGX* (Farrugia, 2012 ▶) and *PLATON* (Spek, 2009 ▶).

## Supplementary Material

Crystal structure: contains datablock(s) I, global. DOI: 10.1107/S1600536813014220/cv5414sup1.cif

Structure factors: contains datablock(s) I. DOI: 10.1107/S1600536813014220/cv5414Isup2.hkl

Supplementary material file. DOI: 10.1107/S1600536813014220/cv5414Isup3.cml

Additional supplementary materials:  crystallographic information; 3D view; checkCIF report

## Figures and Tables

**Table 1:** Hydrogen-bond geometry (Å, °) *Cg*1 is the centroid of the C1–C6 ring.

*D*—H⋯*A*	*D*—H	H⋯*A*	*D*⋯*A*	*D*—H⋯*A*
C17—H17*A*⋯O1^i^	0.96	2.47	3.372 (2)	156
C24—H24*B*⋯*Cg*1^ii^	0.97	2.74	3.624 (2)	152

Symmetry codes: (i) 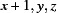; (ii) 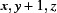.

**Fig. 1.**
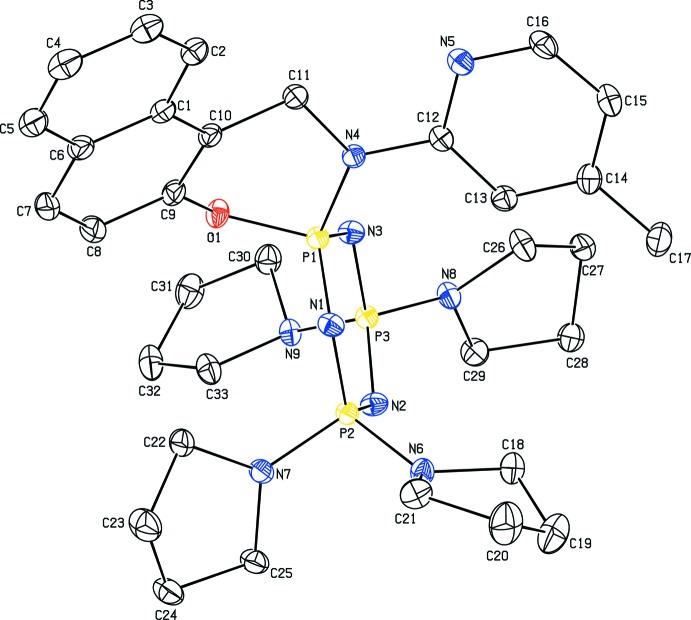
The molecular structure of the title compound with the atom-numbering scheme. Displacement ellipsoids are drawn at the 50% probability level. Hydrogen atoms have been omitted for clarity.

**Fig. 2.**
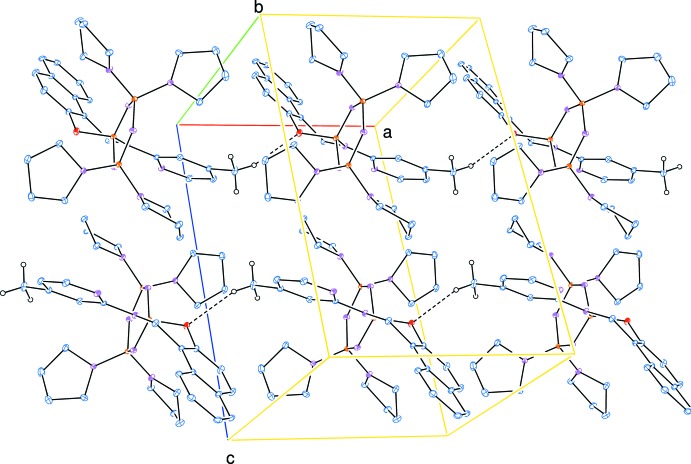
A portion of the crystal packing showing hydrogen bonds as dashed lines. Hydrogen atoms not involved in hydrogen bonding have been omitted for clarity.

## supplementary crystallographic information

### Comment

As a part of our ongoing investigation on the reaction of hexachlorocyclotriphosphazene, N_3_P_3_Cl_6_, with N/O donor type bifunctional reagents such as aminoalcohols (Beşli *et al.*, 2007), aminophenols (Işıklan *et al.*, 2010) and aminonaphthols (Işıklan *et al.*, 2013), the title compound was synthesized and its crystal structure is reported herein.

In the title compound (Fig. 1), the phosphazene ring (*A*) is in flattened-chair conformation [φ = -12.9(1.3)° and θ = 17.5 (3)°] having total puckering amplitude Q_T_ of 0.181 (1) Å (Cremer & Pople, 1975). Atoms N1, N2 and N3 are displaced from the plane through the P atoms by 0.160 (1), 0.081 (1) and 0.184 (1) Å, respectively. As expected, the naphthalene bicycle and the pyridine ring are planar, and they are oriented at a dihedral angle of 41.82 (4)°. Ring *B* (P1/O1/C9—C11/N4) is in twisted-boat conformation [φ = 43.3 (9)° and θ = 31.5 (1)°] having total puckering amplitude Q_T_ of 0.658 (4) Å.

The pyrrolidine rings, *F* (N6/C18—C21), *G* (N7/C22—C25), *H* (N8/C26—C29) and *I* (N9/C30—C33), adopt envelope conformations with atoms C18, C23, C27 and N9 displaced by -0.488 (2), 0.563 (2), -0.606 (2) and -0.557 (1) Å from the planes of the other rings atoms, respectively.

In the phosphazene ring, the P—N bond lengths are in the range of 1.5732 (13)–1.6164 (13) Å [average value is 1.5954 (13) Å], exhibiting a regular variation with distances from P1: P1—N1 ≈ P1—N3 〈 P2—N2 ≈ P3—N2 〈 P2—N1 ≈ P3—N3, and showing double-bond character. However, the exocyclic P1—N4 bond [1.6804 (13) Å] is at the lower limit of the single bond length. In the phosphazene compounds, the P—N and P═N bonds are generally in the ranges of 1.628–1.691 and 1.571–1.604 Å, respectively (Allen *et al.*, 1987). The shortening in the P1—N4 bond is probably due to electron transfer from N4 to the phosphazene ring. On the other hand, the exocyclic P2—N6 [1.6483 (14) Å], P2—N7 [1.6430 (14) Å], P3—N8 [1.6372 (14) Å] and P3—N9 [1.6644 (13) Å] bonds are also at the lower limit of the single bond length.

In the phosphazene ring, the endocyclic N1—P1—N3 angle [118.29 (7)°] is not changed, and the exocyclic O1—P1—N4 angle [99.48 (6)°] is decreased, while the endocyclic N1—P2—N2 [116.01 (7)°] and N2—P3—N3 [115.10 (7)°] angles are decreased with electron donation and withdrawal by the substituents, relative to the 'standard compound' N_3_P_3_Cl_6_ (Bullen, 1971). In the latter compound, the corresponding angles are 118.3, 118.5, 101.2 and 101.6 °, respectively.

The P1—N1—P2, P2—N2—P3 and P1—N3—P3 angles are 120.66 (8), 123.76 (8) and 121.24 (8) °, respectively; P1—N1—P2 is decreased, while P2—N2—P3 is increased with electron donation and withdrawal by the N_3_P_3_ ring. They can be compared with the average value reported for N_3_P_3_Cl_6_, *viz*. 121.4 (3)°.

In the crystal, weak intermolecular C—H···O hydrogen bonds (Table 1) link the molecules related by translation along the *a* axis into chains (Fig. 2), and C—H···π interactions (Table 1) aggregate these chains into layers parallel to *ab* plane.

### Experimental

In a 250 ml three-necked round-bottomed flask, 4',4',6',6'-tetrachloro-2-(4 -methylpyridin-2-yl)-1*H*,2*H*-*spiro*[naphtho[1,2-*e*][1,3,2] oxazaphosphinine-3,2'-[1,3,5,2,4,6]triazatriphosphinine] (1.00 g, 1.86 mmol) was dissolved in dry toluene (100 ml). Six equivalents of pyrrolidine (0.93 ml, 11.20 mmol) and triethylamine (5 ml, 36.00 mmol) were added to the solution. The reaction mixture was refluxed for 12 h. The progress of the reaction was monitored by TLC. The precipitated triethylamine hydrochloride was filtered off. The solvent was evaporated and the product was purified through a silica gel column with a mobile phase of toluene/THF (1:1). The oily product was recrystallized in acetonitrile [m.p. 460 K, yield: 1.16 g, 92%].

### Refinement

H atoms were positioned geometrically with C—H = 0.93, 0.96 and 0.97 Å for aromatic, methyl and methylene H atoms, respectively, and constrained to ride on their parent atoms, with *U*_iso_(H) = k × *U*_eq_(C), where k = 1.5 for methyl H-atoms and k = 1.2 for all other H-atoms.

### Crystal data

**Table:**

C_33_H_46_N_9_OP_3_	*Z* = 2
*M**_r_* = 677.70	*F*(000) = 720
Triclinic, *P*1	*D*_x_ = 1.355 Mg m^−^^3^
Hall symbol: -P 1	Mo *K*α radiation, λ = 0.71073 Å
*a* = 9.5830 (2) Å	Cell parameters from 9939 reflections
*b* = 10.9142 (2) Å	θ = 2.2–28.3°
*c* = 16.8172 (3) Å	µ = 0.22 mm^−^^1^
α = 79.210 (2)°	*T* = 100 K
β = 84.542 (3)°	Block, colourless
γ = 74.193 (2)°	0.31 × 0.26 × 0.25 mm
*V* = 1660.68 (6) Å^3^	

### Data collection

**Table:**

Bruker Kappa APEXII CCD area-detector diffractometer	8270 independent reflections
Radiation source: fine-focus sealed tube	6876 reflections with *I* > 2σ(*I*)
Graphite monochromator	*R*_int_ = 0.030
φ and ω scans	θ_max_ = 28.4°, θ_min_ = 1.2°
Absorption correction: multi-scan (*SADABS*; Bruker, 2005)	*h* = −12→12
*T*_min_ = 0.934, *T*_max_ = 0.947	*k* = −14→14
30003 measured reflections	*l* = −21→22

### Refinement

**Table:**

Refinement on *F*^2^	Primary atom site location: structure-invariant direct methods
Least-squares matrix: full	Secondary atom site location: difference Fourier map
*R*[*F*^2^ > 2σ(*F*^2^)] = 0.040	Hydrogen site location: inferred from neighbouring sites
*wR*(*F*^2^) = 0.109	H-atom parameters constrained
*S* = 1.04	*w* = 1/[σ^2^(*F*_o_^2^) + (0.0556*P*)^2^ + 0.8708*P*] where *P* = (*F*_o_^2^ + 2*F*_c_^2^)/3
8270 reflections	(Δ/σ)_max_ = 0.001
416 parameters	Δρ_max_ = 0.89 e Å^−^^3^
0 restraints	Δρ_min_ = −0.37 e Å^−^^3^

### Special details

**Table:**

Geometry. All e.s.d.'s (except the e.s.d. in the dihedral angle between two l.s. planes) are estimated using the full covariance matrix. The cell e.s.d.'s are taken into account individually in the estimation of e.s.d.'s in distances, angles and torsion angles; correlations between e.s.d.'s in cell parameters are only used when they are defined by crystal symmetry. An approximate (isotropic) treatment of cell e.s.d.'s is used for estimating e.s.d.'s involving l.s. planes.
Refinement. Refinement of *F*^2^ against ALL reflections. The weighted *R*-factor *wR* and goodness of fit *S* are based on *F*^2^, conventional *R*-factors *R* are based on *F*, with *F* set to zero for negative *F*^2^. The threshold expression of *F*^2^ > σ(*F*^2^) is used only for calculating *R*-factors(gt) *etc*. and is not relevant to the choice of reflections for refinement. *R*-factors based on *F*^2^ are statistically about twice as large as those based on *F*, and *R*- factors based on ALL data will be even larger.

### Fractional atomic coordinates and isotropic or equivalent isotropic displacement parameters (Å^2^)

**Table:**

	*x*	*y*	*z*	*U*_iso_*/*U*_eq_	
P1	0.41615 (4)	0.65219 (4)	0.26913 (2)	0.01269 (9)	
P2	0.42634 (4)	0.90846 (4)	0.24147 (2)	0.01288 (9)	
P3	0.36895 (4)	0.78543 (4)	0.39892 (2)	0.01263 (9)	
O1	0.26440 (11)	0.63318 (11)	0.24585 (7)	0.0168 (2)	
N1	0.45484 (14)	0.77140 (12)	0.21130 (8)	0.0147 (3)	
N2	0.40058 (14)	0.90563 (12)	0.33662 (8)	0.0156 (3)	
N3	0.40214 (14)	0.65238 (12)	0.36308 (8)	0.0147 (3)	
N4	0.52927 (14)	0.51427 (12)	0.24700 (8)	0.0140 (3)	
N5	0.74070 (15)	0.34880 (13)	0.26606 (8)	0.0171 (3)	
N6	0.56147 (14)	0.97631 (13)	0.21432 (8)	0.0165 (3)	
N7	0.29558 (15)	1.00713 (12)	0.18643 (8)	0.0174 (3)	
N8	0.45650 (14)	0.74826 (12)	0.48273 (8)	0.0156 (3)	
N9	0.19774 (14)	0.83237 (12)	0.43381 (8)	0.0148 (3)	
C1	0.34522 (17)	0.45778 (15)	0.07336 (9)	0.0153 (3)	
C2	0.44802 (18)	0.35388 (15)	0.04381 (10)	0.0179 (3)	
H2	0.5298	0.3100	0.0730	0.021*	
C3	0.42865 (19)	0.31713 (16)	−0.02734 (10)	0.0211 (3)	
H3	0.4971	0.2487	−0.0457	0.025*	
C4	0.3060 (2)	0.38236 (17)	−0.07261 (10)	0.0234 (4)	
H4	0.2931	0.3564	−0.1204	0.028*	
C5	0.2061 (2)	0.48350 (16)	−0.04650 (10)	0.0230 (4)	
H5	0.1261	0.5267	−0.0773	0.028*	
C6	0.22176 (18)	0.52431 (15)	0.02693 (10)	0.0185 (3)	
C7	0.11652 (19)	0.62697 (16)	0.05555 (11)	0.0229 (4)	
H7	0.0355	0.6698	0.0255	0.027*	
C8	0.13190 (18)	0.66438 (16)	0.12675 (11)	0.0216 (3)	
H8	0.0627	0.7324	0.1451	0.026*	
C9	0.25415 (17)	0.59798 (15)	0.17156 (10)	0.0163 (3)	
C10	0.35985 (16)	0.49761 (14)	0.14797 (9)	0.0146 (3)	
C11	0.48332 (17)	0.42860 (15)	0.20221 (10)	0.0170 (3)	
H11A	0.4544	0.3611	0.2409	0.020*	
H11B	0.5656	0.3876	0.1696	0.020*	
C12	0.67301 (16)	0.47150 (14)	0.27157 (9)	0.0139 (3)	
C13	0.74130 (17)	0.55364 (15)	0.29904 (10)	0.0165 (3)	
H13	0.6903	0.6380	0.3035	0.020*	
C14	0.88477 (17)	0.50805 (16)	0.31940 (10)	0.0182 (3)	
C15	0.95706 (18)	0.38142 (16)	0.31073 (10)	0.0205 (3)	
H15	1.0547	0.3480	0.3219	0.025*	
C16	0.88029 (18)	0.30691 (16)	0.28526 (10)	0.0203 (3)	
H16	0.9287	0.2219	0.2811	0.024*	
C17	0.96028 (19)	0.59051 (18)	0.35269 (12)	0.0263 (4)	
H17A	1.0582	0.5770	0.3303	0.039*	
H17B	0.9613	0.5673	0.4106	0.039*	
H17C	0.9095	0.6798	0.3383	0.039*	
C18	0.68198 (18)	0.95919 (17)	0.26621 (11)	0.0225 (4)	
H18A	0.7359	0.8692	0.2780	0.027*	
H18B	0.6478	0.9906	0.3167	0.027*	
C19	0.7737 (2)	1.0406 (2)	0.21450 (13)	0.0341 (5)	
H19A	0.7362	1.1310	0.2194	0.041*	
H19B	0.8738	1.0115	0.2301	0.041*	
C20	0.7616 (2)	1.0203 (2)	0.12923 (13)	0.0352 (5)	
H20A	0.8378	0.9470	0.1161	0.042*	
H20B	0.7688	1.0964	0.0904	0.042*	
C21	0.61243 (19)	0.99544 (17)	0.12823 (10)	0.0218 (3)	
H21A	0.5465	1.0687	0.0972	0.026*	
H21B	0.6199	0.9191	0.1047	0.026*	
C22	0.20688 (19)	0.97192 (16)	0.13292 (11)	0.0218 (3)	
H22A	0.1234	0.9483	0.1624	0.026*	
H22B	0.2630	0.9008	0.1068	0.026*	
C23	0.1608 (2)	1.09529 (17)	0.07164 (11)	0.0276 (4)	
H23A	0.0726	1.0987	0.0464	0.033*	
H23B	0.2366	1.1024	0.0298	0.033*	
C24	0.13567 (19)	1.20106 (16)	0.12308 (11)	0.0227 (4)	
H24A	0.0379	1.2185	0.1474	0.027*	
H24B	0.1506	1.2802	0.0906	0.027*	
C25	0.24817 (18)	1.14693 (15)	0.18844 (10)	0.0191 (3)	
H25A	0.3293	1.1856	0.1761	0.023*	
H25B	0.2049	1.1631	0.2413	0.023*	
C26	0.60087 (18)	0.65564 (17)	0.49101 (10)	0.0212 (3)	
H26A	0.6708	0.6825	0.4507	0.025*	
H26B	0.5966	0.5696	0.4861	0.025*	
C27	0.63870 (18)	0.66012 (15)	0.57564 (10)	0.0202 (3)	
H27A	0.7429	0.6326	0.5815	0.024*	
H27B	0.5927	0.6061	0.6162	0.024*	
C28	0.57847 (19)	0.80239 (16)	0.58221 (11)	0.0236 (4)	
H28A	0.6430	0.8531	0.5545	0.028*	
H28B	0.5636	0.8144	0.6384	0.028*	
C29	0.43433 (18)	0.83939 (16)	0.54080 (10)	0.0190 (3)	
H29A	0.3554	0.8293	0.5799	0.023*	
H29B	0.4130	0.9281	0.5127	0.023*	
C30	0.13884 (17)	0.73570 (16)	0.49065 (10)	0.0196 (3)	
H30A	0.1860	0.7133	0.5419	0.023*	
H30B	0.1512	0.6578	0.4679	0.023*	
C31	−0.02248 (18)	0.80459 (18)	0.50156 (11)	0.0239 (4)	
H31A	−0.0403	0.8431	0.5503	0.029*	
H31B	−0.0818	0.7444	0.5053	0.029*	
C32	−0.05706 (18)	0.90933 (18)	0.42606 (11)	0.0235 (4)	
H32A	−0.1318	0.8957	0.3962	0.028*	
H32B	−0.0903	0.9944	0.4412	0.028*	
C33	0.08559 (17)	0.89666 (16)	0.37477 (10)	0.0196 (3)	
H33A	0.0893	0.8448	0.3333	0.024*	
H33B	0.0972	0.9808	0.3491	0.024*	

### Atomic displacement parameters (Å^2^)

**Table:**

	*U*^11^	*U*^22^	*U*^33^	*U*^12^	*U*^13^	*U*^23^
P1	0.01320 (18)	0.01268 (18)	0.01255 (19)	−0.00217 (14)	−0.00114 (14)	−0.00448 (14)
P2	0.01397 (19)	0.01263 (18)	0.01200 (19)	−0.00272 (14)	−0.00155 (14)	−0.00256 (14)
P3	0.01362 (18)	0.01335 (18)	0.01120 (19)	−0.00266 (14)	−0.00087 (14)	−0.00389 (14)
O1	0.0142 (5)	0.0201 (6)	0.0184 (6)	−0.0037 (4)	−0.0011 (4)	−0.0098 (5)
N1	0.0180 (6)	0.0132 (6)	0.0123 (6)	−0.0015 (5)	−0.0009 (5)	−0.0040 (5)
N2	0.0210 (7)	0.0139 (6)	0.0131 (6)	−0.0055 (5)	−0.0005 (5)	−0.0037 (5)
N3	0.0188 (6)	0.0135 (6)	0.0120 (6)	−0.0037 (5)	−0.0009 (5)	−0.0033 (5)
N4	0.0147 (6)	0.0130 (6)	0.0153 (6)	−0.0022 (5)	−0.0023 (5)	−0.0058 (5)
N5	0.0189 (7)	0.0166 (6)	0.0150 (7)	−0.0013 (5)	−0.0030 (5)	−0.0041 (5)
N6	0.0165 (6)	0.0201 (7)	0.0134 (7)	−0.0064 (5)	−0.0025 (5)	−0.0008 (5)
N7	0.0195 (7)	0.0136 (6)	0.0194 (7)	−0.0018 (5)	−0.0070 (5)	−0.0041 (5)
N8	0.0150 (6)	0.0164 (6)	0.0146 (7)	0.0001 (5)	−0.0028 (5)	−0.0063 (5)
N9	0.0125 (6)	0.0178 (6)	0.0138 (6)	−0.0026 (5)	−0.0016 (5)	−0.0037 (5)
C1	0.0189 (7)	0.0153 (7)	0.0137 (7)	−0.0079 (6)	−0.0012 (6)	−0.0019 (6)
C2	0.0208 (8)	0.0198 (8)	0.0148 (8)	−0.0078 (6)	−0.0006 (6)	−0.0039 (6)
C3	0.0281 (9)	0.0221 (8)	0.0162 (8)	−0.0103 (7)	0.0025 (7)	−0.0069 (6)
C4	0.0371 (10)	0.0261 (9)	0.0118 (8)	−0.0157 (8)	−0.0021 (7)	−0.0031 (6)
C5	0.0319 (9)	0.0228 (8)	0.0168 (8)	−0.0123 (7)	−0.0103 (7)	0.0025 (6)
C6	0.0238 (8)	0.0163 (7)	0.0173 (8)	−0.0083 (6)	−0.0061 (6)	−0.0003 (6)
C7	0.0234 (8)	0.0187 (8)	0.0270 (9)	−0.0035 (7)	−0.0127 (7)	−0.0022 (7)
C8	0.0189 (8)	0.0165 (8)	0.0294 (9)	−0.0007 (6)	−0.0063 (7)	−0.0072 (7)
C9	0.0170 (7)	0.0172 (7)	0.0171 (8)	−0.0062 (6)	−0.0029 (6)	−0.0054 (6)
C10	0.0162 (7)	0.0147 (7)	0.0145 (8)	−0.0058 (6)	−0.0023 (6)	−0.0031 (6)
C11	0.0198 (8)	0.0145 (7)	0.0182 (8)	−0.0025 (6)	−0.0053 (6)	−0.0072 (6)
C12	0.0148 (7)	0.0158 (7)	0.0101 (7)	−0.0024 (6)	−0.0002 (6)	−0.0023 (6)
C13	0.0169 (7)	0.0157 (7)	0.0168 (8)	−0.0025 (6)	−0.0003 (6)	−0.0051 (6)
C14	0.0165 (7)	0.0231 (8)	0.0154 (8)	−0.0051 (6)	−0.0009 (6)	−0.0046 (6)
C15	0.0154 (7)	0.0247 (8)	0.0195 (8)	−0.0003 (6)	−0.0036 (6)	−0.0052 (7)
C16	0.0200 (8)	0.0175 (8)	0.0203 (8)	0.0023 (6)	−0.0031 (6)	−0.0053 (6)
C17	0.0183 (8)	0.0290 (9)	0.0349 (10)	−0.0065 (7)	−0.0049 (7)	−0.0111 (8)
C18	0.0196 (8)	0.0254 (9)	0.0240 (9)	−0.0067 (7)	−0.0065 (7)	−0.0039 (7)
C19	0.0261 (10)	0.0422 (11)	0.0402 (12)	−0.0167 (9)	0.0005 (8)	−0.0114 (9)
C20	0.0250 (10)	0.0495 (13)	0.0313 (11)	−0.0156 (9)	0.0047 (8)	−0.0021 (9)
C21	0.0243 (8)	0.0238 (8)	0.0178 (8)	−0.0090 (7)	0.0037 (7)	−0.0029 (6)
C22	0.0221 (8)	0.0183 (8)	0.0260 (9)	−0.0033 (6)	−0.0105 (7)	−0.0047 (7)
C23	0.0331 (10)	0.0263 (9)	0.0220 (9)	−0.0028 (8)	−0.0094 (8)	−0.0034 (7)
C24	0.0258 (9)	0.0170 (8)	0.0221 (9)	0.0001 (6)	−0.0063 (7)	−0.0011 (6)
C25	0.0217 (8)	0.0138 (7)	0.0202 (8)	−0.0009 (6)	−0.0039 (6)	−0.0031 (6)
C26	0.0175 (8)	0.0236 (8)	0.0192 (8)	0.0026 (6)	−0.0046 (6)	−0.0052 (7)
C27	0.0217 (8)	0.0179 (8)	0.0207 (8)	−0.0044 (6)	−0.0079 (7)	−0.0002 (6)
C28	0.0277 (9)	0.0209 (8)	0.0246 (9)	−0.0068 (7)	−0.0122 (7)	−0.0038 (7)
C29	0.0225 (8)	0.0188 (8)	0.0166 (8)	−0.0033 (6)	−0.0033 (6)	−0.0069 (6)
C30	0.0171 (8)	0.0234 (8)	0.0184 (8)	−0.0068 (6)	0.0002 (6)	−0.0025 (6)
C31	0.0179 (8)	0.0311 (9)	0.0248 (9)	−0.0079 (7)	0.0033 (7)	−0.0095 (7)
C32	0.0154 (8)	0.0302 (9)	0.0253 (9)	−0.0026 (7)	−0.0041 (7)	−0.0092 (7)
C33	0.0171 (8)	0.0226 (8)	0.0178 (8)	−0.0007 (6)	−0.0043 (6)	−0.0050 (6)

### Geometric parameters (Å, º)

**Table:**

P1—O1	1.6168 (11)	C15—C16	1.379 (2)
P1—N1	1.5801 (13)	C15—H15	0.9300
P1—N3	1.5732 (13)	C16—H16	0.9300
P1—N4	1.6804 (13)	C17—H17A	0.9600
P2—N1	1.6140 (13)	C17—H17B	0.9600
P2—N2	1.5914 (13)	C17—H17C	0.9600
P2—N6	1.6483 (14)	C18—C19	1.519 (3)
P2—N7	1.6430 (14)	C18—H18A	0.9700
P3—N2	1.5975 (13)	C18—H18B	0.9700
P3—N3	1.6164 (13)	C19—H19A	0.9700
P3—N8	1.6372 (14)	C19—H19B	0.9700
P3—N9	1.6644 (13)	C20—C19	1.511 (3)
O1—C9	1.3934 (18)	C20—H20A	0.9700
N4—C11	1.4787 (19)	C20—H20B	0.9700
N4—C12	1.4043 (19)	C21—C20	1.528 (2)
N5—C12	1.3359 (19)	C21—H21A	0.9700
N5—C16	1.340 (2)	C21—H21B	0.9700
N6—C18	1.464 (2)	C22—C23	1.521 (2)
N6—C21	1.479 (2)	C22—H22A	0.9700
N7—C22	1.460 (2)	C22—H22B	0.9700
N7—C25	1.4746 (19)	C23—C24	1.524 (2)
N8—C26	1.476 (2)	C23—H23A	0.9700
N8—C29	1.4823 (19)	C23—H23B	0.9700
N9—C30	1.480 (2)	C24—H24A	0.9700
N9—C33	1.471 (2)	C24—H24B	0.9700
C1—C2	1.419 (2)	C25—C24	1.535 (2)
C1—C6	1.426 (2)	C25—H25A	0.9700
C1—C10	1.433 (2)	C25—H25B	0.9700
C2—C3	1.375 (2)	C26—C27	1.514 (2)
C2—H2	0.9300	C26—H26A	0.9700
C3—C4	1.407 (3)	C26—H26B	0.9700
C3—H3	0.9300	C27—C28	1.523 (2)
C4—C5	1.361 (3)	C27—H27A	0.9700
C4—H4	0.9300	C27—H27B	0.9700
C5—H5	0.9300	C28—H28A	0.9700
C6—C5	1.422 (2)	C28—H28B	0.9700
C6—C7	1.414 (2)	C29—C28	1.526 (2)
C7—C8	1.368 (2)	C29—H29A	0.9700
C7—H7	0.9300	C29—H29B	0.9700
C8—H8	0.9300	C30—C31	1.534 (2)
C9—C8	1.402 (2)	C30—H30A	0.9700
C9—C10	1.366 (2)	C30—H30B	0.9700
C10—C11	1.504 (2)	C31—H31A	0.9700
C11—H11A	0.9700	C31—H31B	0.9700
C11—H11B	0.9700	C32—C31	1.536 (3)
C13—C12	1.406 (2)	C32—C33	1.533 (2)
C13—C14	1.381 (2)	C32—H32A	0.9700
C13—H13	0.9300	C32—H32B	0.9700
C14—C17	1.506 (2)	C33—H33A	0.9700
C15—C14	1.395 (2)	C33—H33B	0.9700
			
N1—P1—O1	110.67 (7)	N6—C18—H18A	111.3
N3—P1—O1	105.79 (6)	N6—C18—H18B	111.3
N3—P1—N1	118.29 (7)	C19—C18—H18A	111.3
O1—P1—N4	99.48 (6)	C19—C18—H18B	111.3
N1—P1—N4	109.64 (7)	H18A—C18—H18B	109.2
N3—P1—N4	111.27 (7)	C18—C19—H19A	111.0
N1—P2—N6	112.35 (7)	C18—C19—H19B	111.0
N1—P2—N7	105.29 (7)	C20—C19—C18	103.98 (15)
N2—P2—N1	116.01 (7)	C20—C19—H19A	111.0
N2—P2—N6	105.52 (7)	C20—C19—H19B	111.0
N2—P2—N7	115.07 (7)	H19A—C19—H19B	109.0
N7—P2—N6	101.83 (7)	C19—C20—C21	105.42 (15)
N2—P3—N3	115.10 (7)	C19—C20—H20A	110.7
N2—P3—N8	115.01 (7)	C19—C20—H20B	110.7
N2—P3—N9	106.61 (7)	C21—C20—H20A	110.7
N3—P3—N8	105.41 (7)	C21—C20—H20B	110.7
N3—P3—N9	112.55 (7)	H20A—C20—H20B	108.8
N8—P3—N9	101.46 (7)	N6—C21—C20	104.84 (14)
C9—O1—P1	119.89 (9)	N6—C21—H21A	110.8
P1—N1—P2	120.66 (8)	N6—C21—H21B	110.8
P2—N2—P3	123.76 (8)	C20—C21—H21A	110.8
P1—N3—P3	121.24 (8)	C20—C21—H21B	110.8
C11—N4—P1	122.37 (10)	H21A—C21—H21B	108.9
C12—N4—P1	121.97 (10)	N7—C22—C23	102.68 (13)
C12—N4—C11	115.65 (12)	N7—C22—H22A	111.2
C12—N5—C16	117.13 (14)	N7—C22—H22B	111.2
C18—N6—P2	123.16 (11)	C23—C22—H22A	111.2
C18—N6—C21	109.75 (13)	C23—C22—H22B	111.2
C21—N6—P2	119.56 (11)	H22A—C22—H22B	109.1
C22—N7—P2	126.69 (11)	C22—C23—C24	103.09 (14)
C22—N7—C25	110.66 (13)	C22—C23—H23A	111.1
C25—N7—P2	122.60 (11)	C22—C23—H23B	111.1
C26—N8—P3	122.62 (11)	C24—C23—H23A	111.1
C26—N8—C29	110.42 (12)	C24—C23—H23B	111.1
C29—N8—P3	121.92 (10)	H23A—C23—H23B	109.1
C30—N9—P3	117.42 (10)	C23—C24—C25	104.67 (13)
C33—N9—P3	118.11 (10)	C23—C24—H24A	110.8
C33—N9—C30	106.12 (12)	C23—C24—H24B	110.8
C2—C1—C6	118.33 (14)	C25—C24—H24A	110.8
C2—C1—C10	122.78 (14)	C25—C24—H24B	110.8
C6—C1—C10	118.89 (14)	H24A—C24—H24B	108.9
C1—C2—H2	119.5	N7—C25—C24	104.50 (13)
C3—C2—C1	120.99 (15)	N7—C25—H25A	110.9
C3—C2—H2	119.5	N7—C25—H25B	110.9
C2—C3—C4	120.48 (16)	C24—C25—H25A	110.9
C2—C3—H3	119.8	C24—C25—H25B	110.9
C4—C3—H3	119.8	H25A—C25—H25B	108.9
C5—C4—C3	120.04 (15)	N8—C26—C27	102.75 (13)
C5—C4—H4	120.0	N8—C26—H26A	111.2
C3—C4—H4	120.0	N8—C26—H26B	111.2
C4—C5—C6	121.26 (16)	C27—C26—H26A	111.2
C4—C5—H5	119.4	C27—C26—H26B	111.2
C6—C5—H5	119.4	H26A—C26—H26B	109.1
C7—C6—C1	119.51 (15)	C26—C27—C28	102.77 (13)
C7—C6—C5	121.58 (15)	C26—C27—H27A	111.2
C5—C6—C1	118.89 (15)	C26—C27—H27B	111.2
C6—C7—H7	119.5	C28—C27—H27A	111.2
C8—C7—C6	121.07 (15)	C28—C27—H27B	111.2
C8—C7—H7	119.5	H27A—C27—H27B	109.1
C7—C8—C9	118.64 (15)	C27—C28—C29	103.16 (13)
C7—C8—H8	120.7	C27—C28—H28A	111.1
C9—C8—H8	120.7	C27—C28—H28B	111.1
O1—C9—C8	117.84 (14)	C29—C28—H28A	111.1
C10—C9—O1	118.51 (14)	C29—C28—H28B	111.1
C10—C9—C8	123.59 (15)	H28A—C28—H28B	109.1
C1—C10—C11	122.09 (13)	N8—C29—C28	104.14 (13)
C9—C10—C1	118.30 (14)	N8—C29—H29A	110.9
C9—C10—C11	119.55 (14)	N8—C29—H29B	110.9
N4—C11—C10	113.62 (12)	C28—C29—H29A	110.9
N4—C11—H11A	108.8	C28—C29—H29B	110.9
N4—C11—H11B	108.8	H29A—C29—H29B	108.9
C10—C11—H11A	108.8	N9—C30—C31	103.53 (13)
C10—C11—H11B	108.8	N9—C30—H30A	111.1
H11A—C11—H11B	107.7	N9—C30—H30B	111.1
N4—C12—C13	121.86 (13)	C31—C30—H30A	111.1
N5—C12—N4	115.69 (13)	C31—C30—H30B	111.1
N5—C12—C13	122.45 (14)	H30A—C30—H30B	109.0
C12—C13—H13	120.3	C30—C31—C32	105.54 (14)
C14—C13—C12	119.45 (14)	C30—C31—H31A	110.6
C14—C13—H13	120.3	C30—C31—H31B	110.6
C13—C14—C15	118.09 (15)	C32—C31—H31A	110.6
C13—C14—C17	121.28 (15)	C32—C31—H31B	110.6
C15—C14—C17	120.60 (15)	H31A—C31—H31B	108.8
C14—C15—H15	120.8	C31—C32—H32A	110.6
C16—C15—C14	118.41 (15)	C31—C32—H32B	110.6
C16—C15—H15	120.8	C33—C32—C31	105.59 (13)
N5—C16—C15	124.42 (15)	C33—C32—H32A	110.6
N5—C16—H16	117.8	C33—C32—H32B	110.6
C15—C16—H16	117.8	H32A—C32—H32B	108.8
C14—C17—H17A	109.5	N9—C33—C32	103.77 (13)
C14—C17—H17B	109.5	N9—C33—H33A	111.0
C14—C17—H17C	109.5	N9—C33—H33B	111.0
H17A—C17—H17B	109.5	C32—C33—H33A	111.0
H17A—C17—H17C	109.5	C32—C33—H33B	111.0
H17B—C17—H17C	109.5	H33A—C33—H33B	109.0
N6—C18—C19	102.41 (14)		
			
N1—P1—O1—C9	66.13 (12)	P2—N6—C18—C19	−179.54 (12)
N3—P1—O1—C9	−164.59 (11)	C21—N6—C18—C19	31.07 (17)
N4—P1—O1—C9	−49.16 (12)	P2—N6—C21—C20	−163.38 (12)
O1—P1—N1—P2	101.22 (9)	C18—N6—C21—C20	−12.73 (18)
N3—P1—N1—P2	−21.01 (13)	P2—N7—C22—C23	−154.25 (13)
N4—P1—N1—P2	−150.02 (9)	C25—N7—C22—C23	28.39 (18)
O1—P1—N3—P3	−102.47 (9)	P2—N7—C25—C24	175.08 (12)
N1—P1—N3—P3	22.19 (13)	C22—N7—C25—C24	−7.43 (18)
N4—P1—N3—P3	150.44 (8)	P3—N8—C26—C27	176.80 (11)
O1—P1—N4—C11	11.22 (13)	C29—N8—C26—C27	21.54 (17)
O1—P1—N4—C12	−167.31 (12)	P3—N8—C29—C28	−152.11 (12)
N1—P1—N4—C11	−104.85 (13)	C26—N8—C29—C28	3.36 (17)
N1—P1—N4—C12	76.62 (13)	P3—N9—C30—C31	−173.12 (11)
N3—P1—N4—C11	122.38 (12)	C33—N9—C30—C31	−38.50 (16)
N3—P1—N4—C12	−56.14 (14)	P3—N9—C33—C32	172.79 (11)
N2—P2—N1—P1	16.28 (12)	C30—N9—C33—C32	38.53 (16)
N6—P2—N1—P1	137.78 (9)	C6—C1—C2—C3	−0.7 (2)
N7—P2—N1—P1	−112.21 (10)	C10—C1—C2—C3	178.79 (15)
N1—P2—N2—P3	−13.23 (13)	C2—C1—C6—C5	0.5 (2)
N6—P2—N2—P3	−138.30 (10)	C2—C1—C6—C7	179.07 (15)
N7—P2—N2—P3	110.29 (10)	C10—C1—C6—C5	−179.04 (14)
N1—P2—N6—C18	−93.15 (14)	C10—C1—C6—C7	−0.4 (2)
N1—P2—N6—C21	53.41 (14)	C2—C1—C10—C9	−179.34 (14)
N2—P2—N6—C18	34.17 (14)	C2—C1—C10—C11	−2.3 (2)
N2—P2—N6—C21	−179.27 (12)	C6—C1—C10—C9	0.1 (2)
N7—P2—N6—C18	154.67 (13)	C6—C1—C10—C11	177.20 (14)
N7—P2—N6—C21	−58.77 (13)	C1—C2—C3—C4	0.1 (2)
N1—P2—N7—C22	10.18 (16)	C2—C3—C4—C5	0.7 (2)
N1—P2—N7—C25	−172.75 (13)	C3—C4—C5—C6	−0.9 (3)
N2—P2—N7—C22	−118.86 (14)	C1—C6—C5—C4	0.3 (2)
N2—P2—N7—C25	58.22 (15)	C7—C6—C5—C4	−178.25 (16)
N6—P2—N7—C22	127.58 (14)	C1—C6—C7—C8	0.6 (2)
N6—P2—N7—C25	−55.35 (14)	C5—C6—C7—C8	179.21 (16)
N3—P3—N2—P2	14.06 (13)	C6—C7—C8—C9	−0.5 (3)
N8—P3—N2—P2	136.91 (9)	O1—C9—C8—C7	−176.89 (15)
N9—P3—N2—P2	−111.50 (10)	C10—C9—C8—C7	0.2 (3)
N2—P3—N3—P1	−18.27 (12)	O1—C9—C10—C1	177.07 (13)
N8—P3—N3—P1	−146.11 (9)	O1—C9—C10—C11	−0.1 (2)
N9—P3—N3—P1	104.15 (10)	C8—C9—C10—C1	0.0 (2)
N2—P3—N8—C26	−90.19 (14)	C8—C9—C10—C11	−177.17 (15)
N2—P3—N8—C29	62.30 (14)	C1—C10—C11—N4	147.90 (14)
N3—P3—N8—C26	37.71 (14)	C9—C10—C11—N4	−35.1 (2)
N3—P3—N8—C29	−169.80 (12)	C14—C13—C12—N4	−177.42 (14)
N9—P3—N8—C26	155.21 (13)	C14—C13—C12—N5	1.6 (2)
N9—P3—N8—C29	−52.31 (13)	C12—C13—C14—C15	0.8 (2)
N2—P3—N9—C30	177.45 (11)	C12—C13—C14—C17	−177.17 (15)
N2—P3—N9—C33	48.27 (13)	C16—C15—C14—C13	−2.4 (2)
N3—P3—N9—C30	50.35 (13)	C16—C15—C14—C17	175.60 (16)
N3—P3—N9—C33	−78.82 (13)	C14—C15—C16—N5	1.8 (3)
N8—P3—N9—C30	−61.84 (12)	N6—C18—C19—C20	−36.92 (19)
N8—P3—N9—C33	168.98 (11)	C21—C20—C19—C18	29.8 (2)
P1—O1—C9—C8	−134.76 (13)	N6—C21—C20—C19	−11.1 (2)
P1—O1—C9—C10	47.98 (18)	N7—C22—C23—C24	−37.58 (18)
P1—N4—C11—C10	25.90 (18)	C22—C23—C24—C25	33.58 (18)
C12—N4—C11—C10	−155.48 (13)	N7—C25—C24—C23	−16.68 (18)
P1—N4—C12—N5	165.83 (11)	N8—C26—C27—C28	−37.69 (16)
P1—N4—C12—C13	−15.1 (2)	C26—C27—C28—C29	40.19 (17)
C11—N4—C12—N5	−12.79 (19)	N8—C29—C28—C27	−26.81 (17)
C11—N4—C12—C13	166.32 (14)	N9—C30—C31—C32	22.81 (17)
C16—N5—C12—N4	176.79 (14)	C33—C32—C31—C30	−0.03 (18)
C16—N5—C12—C13	−2.3 (2)	C31—C32—C33—N9	−22.92 (17)
C12—N5—C16—C15	0.6 (2)		

### Hydrogen-bond geometry (Å, º)

Cg1 is the centroid of the C1–C6 ring.

**Table:**

*D*—H···*A*	*D*—H	H···*A*	*D*···*A*	*D*—H···*A*
C17—H17*A*···O1^i^	0.96	2.47	3.372 (2)	156
C24—H24*B*···*Cg*1^ii^	0.97	2.74	3.624 (2)	152

Symmetry codes: (i) *x*+1, *y*, *z*; (ii) *x*, *y*+1, *z*.
